# Supplementary material for: Human Adipose Tissue-Derived Stromal Cells Suppress Human, but Not Murine Lymphocyte Proliferation, via Indoleamine 2,3-Dioxygenase Activity
Source: Cells. 2020 Nov 5;9(11):2419. doi: 10.3390/cells9112419 (PMC7694333; doi:10.3390/cells9112419)
Supplement: Supplementary file 1 [file cells-09-02419-s001.pdf]

Supplementary document 1

Extracellular vesicle isolation and characterisation

Preparation of extracellular vesicle (EV)-depleted AB serum. EV-depleted serum was obtained by ultracentrifuging regular AB serum at 4°C, at 100,000g for 18 hours in the WX Ultra Series 100 ultracentrifuge (Thermo Fisher Scientific, Waltham; USA) using a fixed angle rotor Sorvall T-865 (k-factor 100,000g: 150.6) (Thermo Fisher Scientific). Prior to ultracentrifugation, all polycarbonate ultracentrifuge tubes (Beranek Laborgeräte, Weinheim; Germany) were precisely weighed with a precision scale allowing a difference in weight of only 0.01g in order to avoid any possible imbalance. Consequently, ultracentrifuge EV-depleted serum (UC-dserum) was aliquoted and cryopreserved at -30°C for use in future experiments.

EV isolation. $1.5x10^6$ ASC were seeded in a 875 cm^2 5-layer multiflask (Falcon), in DMEM, supplemented with 10% AB serum (German Red Cross Blood Donor Service, Mannheim), 1% Penicillin/Streptomycin (PAN Biotech, Aidenbach; Germany) and 2% L-glutamine (200 M; PAN Biotech), and cells were allowed to attach overnight (ON). The next day media was changed to 100ml DMEM supplemented with 10% UC-d AB serum (depletion of diluted serum by ON centrifugation at 100,000xg), 1% Penicillin/Streptomycin (PAN Biotech) and 2% L-glutamine (200mM; PAN Biotech) and according to the experiments, IFNγ was added to the culture (final concentration 10ng/ml). AB serum supplemented medium was chosen as it promoted high cell expansion and improved EV yield. 72 hours later, CM was collected in 50 ml tubes and proceeded with the EV isolation procedure. The cells were thoroughly washed with PBS, trypsinised and counted to monitor the exact producer cell number present. Cell viability in all cases was higher than 95% as measured by Casy Counter.

EV isolation and purification was performed by consecutive steps of differential ultracentrifugation [1]. CM was centrifuged at RT during 5 minutes at 1,600rpm. Supernatants were retrieved and filtered through a 0.22µm syringe filter in order to eliminate larger cell rests, apoptotic bodies and debris. After filtration, ultracentrifuge tubes were filled with CM and weighed accordingly with a precision scale. Tubes were loaded in WX Ultra Series 100 ultracentrifuge (Thermo Fisher Scientific) using a fixed angle rotor Sorvall T-865 (Thermo Fisher Scientific) with a first UC of 10,000g (k-factor 10,000g: 1569.6; 11,800rpm) at 10°C during 45 minutes. Following the first ultracentrifugation step, supernatants were collected from the original tube and transferred to new sterilized UC tubes for the next UC step. UC tubes were weighed and placed in the ultracentrifuge accordingly, to perform the second UC step of 105,000g (k-factor 105,000g: 150.6; 38,100rpm) at 10°C during 45 minutes. EV pellets were always resuspended in sterile filtered PBS and adjusted to yield 200µl per $2x10^7$ producer cells and stored in 1.5ml low adhesive tubes (Biozym Scientific; Hessisch Oldendorf, Germany) at -30°C for a maximum of 6 months. A small aliquot of supernatant from the second ultracentrifugation step was stored to use as a control in WB analysis or transmission electron microscopy (TEM).

Nanoparticle tracking analysis measurement. One microliter of concentrated EV were diluted in 0.22µm sterile-filtered PBS in a 1:1,000 dilution, EV were visualized using the ZetaView (Particle Metrix, Meersbusch; Germany). The device specific configuration was as follows: 80% sensitivity, shutter 100, 11 positions were measured and two measurement cycles were performed. Data were exported as a PDF report and txt files and further analyzed with GraphPad Prism 7 software (GraphPad Software, California; USA).

Transmission electron microscopy. Five microliters of EV suspensions were left to settle on 100 mesh formvar-coated nickel grids (Plano, Wetzler; Germany), contrasted with 4% uranyl acetate (Serva, Heidelberg; Germany) as a negative staining, air-dried and visualized using a EM10A transmission microscope (Carl Zeiss, Oberkochen; Germany) equipped with a CCD Olympus mega view G2 digital camera (Olympus Soft Imaging Solutions GmbH, Münster; Germany) at 60 KV.

Flow cytometry detection. Following isolation and purification, EV were coupled to aldehyde/sulfate latex beads (Invitrogen, Massachusetts; USA) to allow their better detection in the FACS due to their small size range. 5µl of beads were mixed with 100µl of PBS to pre-wash the beads, then incubated at room temperature for 15-30 minutes at 800rpm in a horizontal rotor. EV were added at this point and 400µl of PBS were added prior to incubation at room temperature for 60 minutes at 800rpm in a horizontal rotor. 400µl of 1M glycine (Serva) (previously filtered through 0.22µm sterile syringe filter) were added and incubated for 60 minutes at 800rpm. Samples were centrifuged for 2 minutes at 12,000rpm and supernatant was discarded leaving no more than 20µL of volume in the tube. Pellets were resuspended in 100µl of 10% BSA solution (Carl Roth) and incubated at RT for 45 minutes at 800rpm in a horizontal rotor. Samples were centrifuged and resuspended in 40µl of 2% BSA solution (Carl Roth) (0.22µm filtered). To assess antigen expression, samples were incubated overnight at 4ºC at 800rpm in a horizontal shaker with the appropriate combination of monoclonal antibodies. The antibodies were: CD9 PerCP-Cy 5.5 (clone M-L13BD; Biosciences, Heidelberg; Germany), CD63 Brilliant violet 421 (clone H5C6; Biolegend), CD81 PE/Cy7 (clone 5A6; Biolegend), TSG101 (clone C-2) and Calnexin (clone AF18) Alexa fluor 647 and Alix PE (Santa Cruz).

Following incubation time, three consecutive washing steps were performed with 200µl of 2% BSA solution. Prior to measurement, 300µl of PBS were added and measured immediately at BD FACSCanto II (BD Biosciences). Unstained beads were used as a negative control, together with beads that were stained in the same manner as the EV samples. EV that were not stained with any antibody were used to discriminate positive and negative population signals.

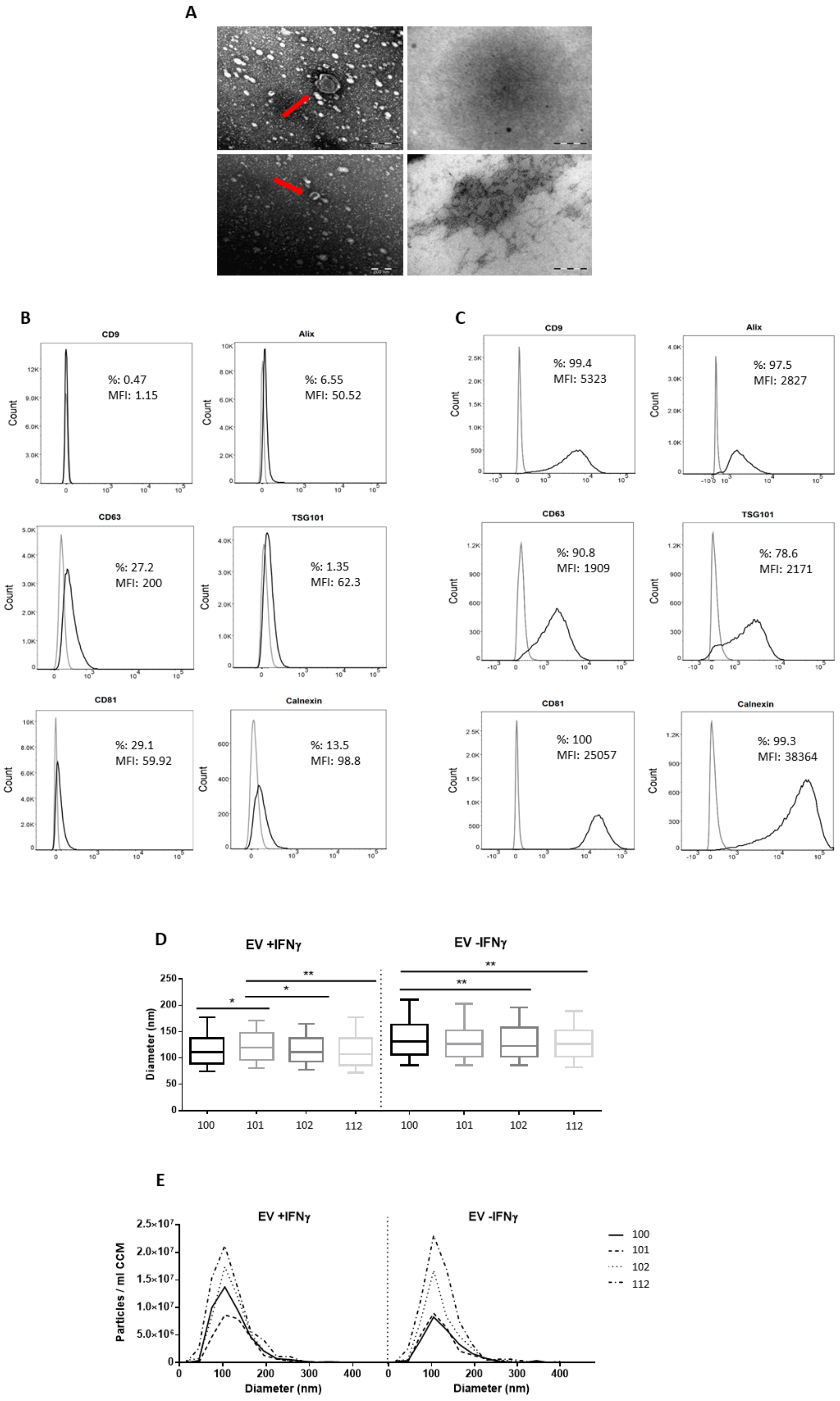
A
B
CD9
%: 0.47
MFI: 1.15
Alix
%: 6.55
MFI: 50.52
CD63
%: 27.2
MFI: 200
TSG101
%: 1.35
MFI: 62.3
CD81
%: 29.1
MFI: 59.92
Calnexin
%: 13.5
MFI: 98.8
Count
C
CD9
%: 99.4
MFI: 5323
Alix
%: 97.5
MFI: 2827
CD63
%: 90.8
MFI: 1909
TSG101
%: 78.6
MFI: 2171
CD81
%: 100
MFI: 25057
Calnexin
%: 99.3
MFI: 38364
D
EV +IFNγ
EV -IFNγ
Diameter (nm)
100
101
102
112
E
EV +IFNγ
EV -IFNγ
Particles / ml CCM
Diameter (nm)
100
101
102
112

Supplementary Figure 1 (A) TEM analysis documents the spherical and cup-shaped EV (red arrows) isolated from different ASC donors (top and bottom left); In the PBS control (top right) no particles are observed. Likewise, in the control EV media (bottom right) traces of protein aggregates but no EV particles are seen. Scale bar 200 nm, representative images shown. (B, C) Flow cytometry of ASC-derived EV (B) and ASC: (C) The relative fluorescence/marker intensity of ASC-derived EV isolates (black line) against unstained EV particle control (light grey line) is depicted in (B) and for ASC against the unstained control in (C) (extracellular staining for CD9, CD63 and CD81 and intracellular staining for Alix, TSG101 and Calnexin). ASC-derived EV were positive for the transmembrane/GPI-anchored proteins CD63 and CD81, but not CD9, weakly positive for cytosolic proteins recovered with EV such as Alix and TSG101, and weakly positive for the negative control ER marker calnexin. ASC ae positive for all markers. (D, E) NTA characterisation of EV. (D) NTA size profile (diameter) of isolated EV +IFNγ and – IFNγ. n=4. NTA analysis showed EV size medians of 115.1 ± 1.8 nm (-IFNγ), 111.1 ± 2.2 nm (+IFNγ), in EV isolates. Box: interquartile range; whiskers: 10th and 90th percentile; line: median. Lines with asterisks depict the significance between two conditions. (E) Full size profiles (yield/ml cell CM against particle size) for EV isolated from +IFNγ pre-stimulated and not (-IFNγ) pre-stimulated MSC. EV counts differed significantly between the four donors (EV +IFNγ: donor 101 vs 112, $p<0.01$; EV -IFNγ: 100 vs 112 and 101 vs 112, $p<0.01$, 2-way ANOVA) (Figure 15 B). However, IFNγ pre-stimulation did not alter the EV yield significantly (n.s.). Irrespective of results of NTA analysis, EV pellets were adjusted to cell equivalents.

(A. Torres Crigna 2020, doctoral thesis. "Comparative analysis of the immunomodulatory properties of different mesenchymal stromal cells and their extracellular vesicles" Medical Faculty Mannheim, Heidelberg University).

REFERENCES

1. Torres Crigna, A.; Fricke, F.; Nitschke, K.; Worst, T.; Erb, U.; Karremann, M.; Buschmann, D.; Elvers-Hornung, S.; Tucher, C.; Schiller, M., et al. Inter-Laboratory Comparison of Extracellular Vesicle Isolation Based on Ultracentrifugation. *Transfus Med Hemother* **2020**, 10.1159/000508712, doi:10.1159/000508712.
